# Safety and bactericidal efficacy of cold atmospheric plasma generated by a flexible surface Dielectric Barrier Discharge device against *Pseudomonas aeruginosa *in vitro and in vivo

**DOI:** 10.1186/s12941-020-00381-z

**Published:** 2020-08-19

**Authors:** Gabrielle S. Dijksteel, Magda M. W. Ulrich, Marcel Vlig, Ana Sobota, Esther Middelkoop, Bouke K. H. L. Boekema

**Affiliations:** 1 Association of Dutch Burn Centres, Zeestraat 29, 1941 AJ Beverwijk, The Netherlands; 2 Dept. of Plastic, Reconstructive & Hand Surgery, Amsterdam Movement Sciences, Amsterdam UMC, Vrije Universiteit Amsterdam, De Boelelaan 1117, 1081 HV Amsterdam, The Netherlands; 3 Dept. of Pathology, Amsterdam UMC, Vrije Universiteit Amsterdam, Amsterdam, The Netherlands; 4 Dept. of Applied Physics, Eindhoven University of Technology, PO Box 513, 5600 MB Eindhoven, The Netherlands

**Keywords:** Dielectric barrier discharge, Cold atmospheric plasma, Efficacy, Safety, *Pseudomonas aeruginosa*, Wound and soft tissue infections

## Abstract

**Background:**

Cold atmospheric plasma (CAP), which is ionized gas produced at atmospheric pressure, could be a novel and potent antimicrobial therapy for the treatment of infected wounds. Previously we have shown that CAP generated with a flexible surface Dielectric Barrier Discharge (sDBD) is highly effective against bacteria in vitro and in ex vivo burn wound models. In the current paper, we determined the in vitro and in vivo safety and efficacy of CAP generated by this sDBD device.

**Methods:**

The effect of CAP on DNA mutations of V79 fibroblasts was measured using a hypoxanthine–guanine-phosphoribosyltransferase (HPRT) assay. Furthermore, effects on cell proliferation, apoptosis and DNA damage in ex vivo burn wound models (BWMs) were assessed using immunohistochemistry. Next, 10^5^ colony forming units (CFU) *P. aeruginosa strain* PAO1 were exposed to CAP in a 3D collagen-elastin matrix environment to determine the number of surviving bacteria in vitro. Finally, rat excision wounds were inoculated with 10^7^ CFU PAO1 for 24 h. The wounds received a single CAP treatment, repeated treatments on 4 consecutive days with CAP, 100 µL of 1% (wt/wt) silver sulfadiazine or no treatment. Wound swabs and punch biopsies were taken to determine the number of surviving bacteria.

**Results:**

Exposure of V79 fibroblasts to CAP did not increase the numbers of mutated colonies. Additionally, the number of proliferative, apoptotic and DNA damaged cells in the BWMs was comparable to that of the unexposed control. Exposure of PAO1 to CAP for 2 min resulted in the complete elimination of bacteria in vitro. Contrarily, CAP treatment for 6 min of rat wounds colonized with PAO1 did not effectively reduce the in vivo bacterial count.

**Conclusions:**

CAP treatment was safe but showed limited efficacy against PAO1 in our rat wound infection model.

## Background

Severely burned patients are at high risk of wound colonization with opportunistic bacteria such as *Staphylococcus aureus* and *Pseudomonas aeruginosa* due to large wound areas and a compromised host defense system [1]. Treatment of infected burns remains a challenge due to the emergence of antibiotic-resistant and persistent bacteria [2]. Additionally, current topical treatments for colonized and infected burns display sub-optimal bactericidal efficacy and may impair wound healing [3–5]. Therefore, novel antimicrobial therapies are needed for the treatment of wound colonization and infection.

A potential antimicrobial therapy to limit bacterial colonization is ionized gas, known as plasma. Plasma is the fourth state of matter in physics and consists of a mix of ions, electrons, highly reactive molecules, excited species, electric fields and ultraviolet radiation [6]. It can be artificially generated by subjecting a neutral gas to an extremely high temperature or a strong electromagnetic field. Often, plasma is accompanied by the production of heat due to the collision of electrons, and the subsequent excitation, ionization and dissociation processes of the gas particles [6].

In the medical field, plasma has been shown to be effective for sterilization, skin resurfacing and coagulation purposes [7–9]. However, these plasmas are generated in vacuum or are extremely hot, making them unsuitable for the treatment of (infected) burns. An alternative approach to treat colonized or infected tissue is cold atmospheric plasma (CAP) [10, 11]. CAP devices generally consist of a powered electrode and a ground electrode of stainless-steel wire mesh. They operate under atmospheric pressure and preferably do not raise the temperature above 40 °C. Temperature rise can be further limited by applying CAP in a pulsed mode [6].

**Fig. 1:**
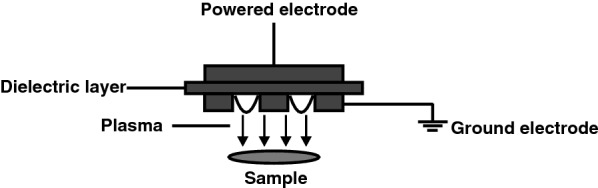
Schematic representation of the generation of CAP by sDBD (Modified from ref [12])

In the current study, we investigated the safety and efficacy of a CAP source called the flexible surface Dielectric Barrier Discharge (sDBD). This plasma source consists of a dielectric plate that separates the powered electrode from the ground electrode, resulting in the formation of gas plasma on the ground electrode (Fig. 1). An advantage of this plasma source is that its use is not limited to small or flat surfaces. Previously, we have shown that CAP generated with this device has excellent bactericidal properties and has no effect on the re-epithelialization process of ex vivo human burn wound models (BWMs) [12]. The current study describes additional efficacy and safety tests. We investigated potential deoxyribonucleic acid (DNA) damage and mutagenesis upon exposure to CAP in vitro. Thereafter, in vivo experiments using rat excision wounds were performed to determine the efficacy of CAP against *P. aeruginosa* in these circumstances.

## Methods

### Plasma source

The flexible sDBD consists of a polyimide (100 μm thick) dielectric barrier strip [12]. The strip has a diameter of 2.5 cm and is integrated into a holder for research purposes. It was operated at 7 kHz, 850–900 mA, 0.032 V_rms_ for up to 6 min at atmospheric pressure in air. The surface between the strip and the treated sample was set at 4 mm and closed from the surroundings to achieve an optimal CAP effluent. The temperature of the samples was measured using a thermal imaging camera FLIR One (FLIR Systems, Inc., Wilsonville, OR, USA) attached to an iPad mini (Apple Inc., Cupertino, CA, USA).

### Cell culture

Chinese Hamster V79 fibroblasts [13] were routinely cultured on tissue culture plastic in Dulbecco's Modified Eagle Medium (DMEM) supplemented with 10% (v/v) fetal calf serum (FCS), 1% (v/v) penicillin/streptomycin (P/S) and 1% Glutamax (all from Gibco, Paisley, UK) further referred to as fibroblast medium (FBM), at 37 °C and 5% CO_2_.

To mimic the in vivo environment, collagen-elastin matrices (Matriderm^®^; MedSkin Solutions Dr. Suwelack AG, Billerberck, Germany) with a diameter of 15 mm and a thickness of 1 mm were used. Matriderm^®^ scaffolds were soaked in FBM, the FBM medium was removed and V79 fibroblasts were seeded (2300 cells/ mm^2^) onto these scaffolds. After 2 h incubation at 37 °C and 5% CO_2_, 2 mL of FBM was added to the scaffolds. The scaffolds were incubated overnight. Prior to exposure, V79 fibroblasts in Matriderm^®^ were washed twice using sterile saline.

### Cell viability

To determine the activity of V79 fibroblasts in Matriderm^®^, 2 mL of resazurin (Merck KGaA, Darmstadt, Germany) with a final concentration of 75 µM in FBM was added to the scaffolds. After 3 h incubation at 37 °C and 5% CO_2_, the fluorescence of the medium (100 µL) was measured using the SpectraMax M2 (Molecular Devices, California, USA) at an excitation and emission wavelength of 540 nm and 595 nm, respectively. To estimate the number of cells, we determined the amount of double-stranded DNA (dsDNA) of the same samples. Resazurin was discarded and 2 mL of 0.05% (v/v) Triton X-100 (Merck KGaA) was added to the cells. Three freeze and thaw cycles were performed at − 80 and 37 °C to lyse the cells. Fifty µL of PicoGreen solution (Quant-iT PicoGreen dsDNA reagent kit, Molecular Probes, Eugene, USA) was added to 50 µL of the samples in a 96-well plate. The fluorescence was measured using the SpectraMax M2 at an excitation and emission wavelength of 480 nm and 520 nm, respectively. Values were interpolated into the standard dsDNA curve for quantification of the number of cells.

### DNA mutation

To determine possible mutations in the DNA of V79 fibroblasts, a modified hypoxanthine–guanine-phosphoribosyltransferase (HPRT) protocol of Davies et al. [14] was used. After exposure of V79 fibroblasts in Matriderm^®^ to CAP, the scaffolds were incubated in 2 mL of FBM overnight at 37 °C and 5% CO_2_. Cells were isolated as follows: scaffolds were incubated with 300 µL of 0.25% (wt/v) collagenase and dispase (Gibco) for 10–15 min at 37 °C and 5% CO_2_. To neutralize collagenase and dispase, 5 mL of 1 mM ethylenediaminetetraacetic acid (EDTA; Gibco-BRL Life Technologies, N.Y., USA) in sterile phosphate-buffered saline (PBS; Gibco) was added to the suspension. The suspension was filtered using a 70 µm cell strainer. After centrifugation at 180*g* for 10 min, V79 fibroblasts were sub-cultured in FBM at 10 cells/cm^2^ (equivalent to 100 cells) to assess the plating efficiency and at 1300 cells/ cm^2^ (equivalent to 10^5^ cells) to estimate the mutation frequency. After 5 days, the FBM of the cell cultures for the estimation of mutation frequency was supplemented with 20 µL of 5 µg/mL 6-thioguanine (6-TG; Merck KGaA). This was added daily during a subsequent 10 days culture. Colonies were stained using crystal violet (Klinipath, Duiven, the Netherlands) and counted microscopically using NIS Elements (Nikon Instruments Europe B.V., Amstelveen, the Netherlands). As positive controls, V79 fibroblasts in Matriderm^®^ were exposed to the mutagenic compound ethyl methanesulfonate (EMS) at a concentration of 0.8 µL of 0.3 mg/mL for 3 h. Negative controls were prepared by washing V79 fibroblasts in Matriderm^®^ with sterile saline.

### Ex vivo wound healing using BWMs

Excess human skin was obtained from patients undergoing elective surgery at the Red Cross Hospital (Beverwijk, the Netherlands) according to institutional guidelines and medical research “code of conduct for responsible use”, drafted by Federa (Foundation Federation of Dutch Medical Scientific Societies). Skin grafts with a thickness of 0.5 mm were prepared using a dermatome (Aesculap AG & Co. KG, Tuttlingen, Germany). A scalpel was used to cut the skin graft into pieces of 1 cm^2^. Burn wounds were inflicted by placing a soldering iron (10 × 2 mm) set at 95 °C on the epidermis for 10 s without exerting pressure [15].

BWMs were cultured air-exposed on stainless-steel grids at 37 °C and 5% CO_2_ using DMEM/ Ham’s F12 (3:1) supplemented with 2% (v/v) P/S, 2% (v/v) FCS (Gibco), 1 µM hydrocortisone, 1 µM isoproterenol, 0.1 µM insulin, 1 µM l-carnitine, M l-serine, 1 µM dl-alpha-tocopherol, 130 µg/mL ascorbic acid, a lipid supplement containing 25 µM palmitic acid, 15 µM linoleic acid, 7 µM arachidonic acid (all from Merck KGaA) and 24 µM bovine serum albumin (Thermo Fisher Scientific, Paisley, UK) [16]. The culture medium was refreshed twice a week during 2 weeks culture. Twenty-four hours before fixation of the BWMs in kryofix (50% ethanol and 3% PEG300), 20 µM 5-bromo-2-deoxyuridine (BrdU; Merck KGaA) was added to the culture medium.

### Immunohistochemistry

Tissue samples were dehydrated and embedded in paraffin. Five µm sections of the paraffin embedded samples were deparaffinized and rehydrated for staining with antibodies: BrdU (B5002, Merck KGaA), Caspase-3 (ab4051, Abcam, Cambridge, UK) and gamma-H2A histone family member X (γH2AX; MA1-2022, Thermo Fisher Scientific) to determine cell proliferation, apoptosis and DNA damage, respectively.

Antigen retrieval was performed using 2 M HCl at room temperature, which was neutralized using 0.1 M Borax (pH 8.5) followed by 0.5% (v/v) Triton X-100 (Merck KGaA) in PBS for BrdU, 10 mM Tris–EDTA at 70 °C for Caspase-3 and 10 mM sodium citrate solution (pH 6) at 65 °C for γH2AX. Powervision polymeric horseradish peroxidase anti Rabbit (Klinipath) and 3,3′-diaminobenzidine substrate (Immunologic, Duiven, The Netherlands) were used for visualization of the BrdU or Caspase-3 positive cells. To detect γH2AX, a fluorescently labelled goat anti-mouse antibody (AF-555, Molecular Probes) and 4′,6′-diamidino-2-fenylindool (Merck KGaA) was used.

Negative controls were performed in absence of the first antibody. All sections stained for BrdU and Caspase-3 were counterstained using hematoxylin and were dehydrated and mounted using Entellan (Merck KGaA). γH2AX stained sections were aqueously mounted. NIS Elements (Nikon Instruments Europe B.V) was used to microscopically measure the newly formed epidermis (outgrowth) and the number of positively stained cells in this area.

### Bacterial culture

A mid-log growth culture of a *P. aeruginosa* strain, PAO1 (ATCC BAA47), was prepared in Luria Bertani (LB) medium at 37 °C, which was shaken at 200 rpm for approximately 3 h. After centrifugation of the bacterial suspension at 3600×*g* for 5 min, the pellet was resuspended in sterile saline to the required concentration, based on the optical density of the bacterial culture at 600 nm.

### In vitro efficacy test

To determine the efficacy of gas plasma in a biologically relevant environment, Matriderm^®^ scaffolds were soaked in sterile saline, inoculated with 10 µL of 1 × 10^7^ colony forming units (CFU)/mL PAO1 for 30 min at room temperature and then exposed to CAP. Thereafter, the scaffolds were transferred to polypropylene vials containing a metal bead and 1 mL of PBS. After homogenizing the samples using a TissueLyser (Qiagen, Venlo, the Netherlands) set at 50 Hz for 4 min, tenfold serial dilutions of the homogenates were prepared. Dilutions were cultured on LB agar plates to quantify the number of viable bacteria after an overnight incubation at 37 °C and 5% CO_2_.

### In vivo efficacy of CAP in a rat model

The experimental protocol for the study of CAP was approved by the Central Authority for Scientific Procedures on Animals (protocol AVD114002016601), according to governmental and international guidelines for animal experimentation.

Twelve male and 12 female rats (Wistar) of 8 to 10 weeks old and a minimum weight of 160 g were purchased from Envigo (Horst, the Netherlands). The animals were acclimatized for 2 weeks prior to wounding. The animals were kept under specific pathogen-free conditions and were housed in individually ventilated cages with tap water and an irradiation-sterilized pelleted diet ad libitum. Wood- shavings were used as bedding material and long paper strips were used as enrichment.

The sample size calculation and detailed experimental procedure for antimicrobial efficacy tests using a rat excision wound model were previously described [17]. To minimize the number of experimental animals, two partial thickness excision wounds of approximately 1 cm^2^ large and 2 cm apart were prepared on the back of the rats using a dermatome set at 0.7 mm. The wounds were equally divided into four treatment groups. Each group had 12 wounds, i.e. one wound on 6 male and 6 female rats.

The wounds were inoculated with 100 µL of 10^8^ CFU/mL PAO1 at t = 0. Twenty-four h after inoculation, the wounds received no treatment (group 1) or a single CAP treatment (group 2) on day 1. Wounds in group 3 and group 4 received repeated treatments on 4 consecutive days with CAP or 100 µL of 1% (wt/wt) silver sulfadiazine in cetomacrogol cream (group 4; SSD; Pharmacy of the Medical Center Alkmaar, Alkmaar, the Netherlands), respectively. To determine the bacterial load, wound swabs were taken before and after CAP treatment. The untreated wounds were swabbed twice on day 1 to assess the effect of swabbing on the bacterial load of the wound. Wound swabs were taken only before SSD treatment to prevent the removal of this topical.

Six male and 6 female rats were euthanized on day 3 and on day 7 using saturated CO_2_/O_2_ followed by CO_2_ only. Four mm punch biopsies were taken from the wounds of the euthanized rats to determine the bacterial load within the tissue.

Samples were homogenized in 1 mL of PBS (Gibco) using a TissueLyser set at 50 Hz for 4 min. Ten-fold serial dilutions of the homogenates were plated on LB agar and *Pseudomonas* isolation agar supplemented with cetrimide (50 mg/L) and sodium nalidixate (3.8 mg/L) (Oxoid ltd, Basingstoke, UK) to selectively identify *P. aeruginosa* from commensal bacteria. After overnight incubation of the plates at 37 °C and 5% CO_2_, the number of viable bacteria was determined.

### Statistical analysis

Statistically significant differences were determined using SPSS version 24. For differences between groups the Kruskal–Wallis test followed by the Mann–Whitney-U test were used. To compare two related groups, the Wilcoxon singed rank sum test was used.

## Results

### In vitro efficacy of CAP against *P. aeruginosa*

**Fig. 2:**
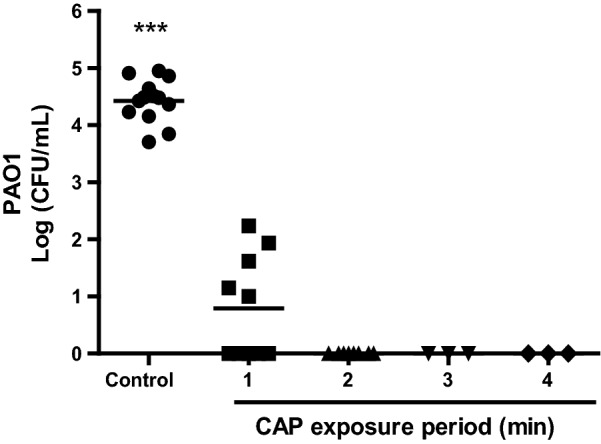
In vitro antibacterial efficacy of CAP. Matriderm^®^ scaffolds inoculated with approximately 10^5^ CFU PAO1 for 30 min were exposed to CAP for 1–4 min. Results are expressed as the number of surviving bacteria in log10 (CFU/mL) versus the exposure period to CAP. Data represent the means of at least three independent experiments performed in duplicate. Statistical differences compared to CAP-exposed samples are indicated: *p < 0.05; **p < 0.01; ***p < 0.001 (MWU)

To determine the optimal exposure time to CAP for an effective bactericidal elimination under the same conditions as in the in vitro safety tests, 10^5^ CFU of PAO1 in Matriderm^®^ were exposed to CAP for 1–4 min. After exposure to CAP for 1 min, 6.2 CFU/mL of PAO1 survived on average (Fig. 2). Exposure to CAP for 2 min or longer resulted in no surviving bacteria.

### Effect of CAP on the viability of V79 fibroblasts

**Fig. 3:**
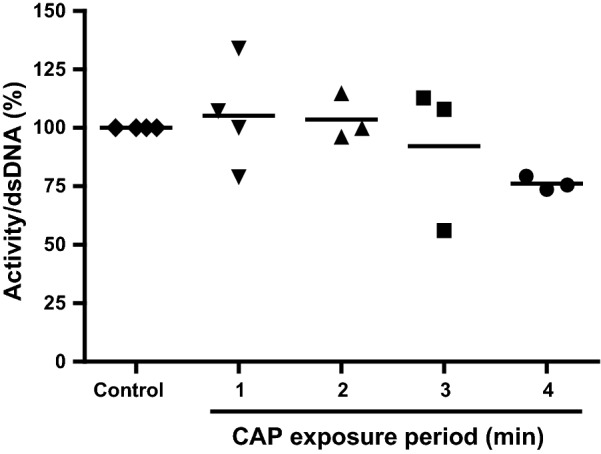
Viability of V79 fibroblasts after exposure to CAP. V79 fibroblasts in Matriderm^®^ were exposed to CAP for 1–4 min and thereafter the metabolic activity per V79 fibroblast was determined. Results are expressed relative to the unexposed control samples. Data represent the means of at least three independent experiments performed in duplicate. Statistically significant differences were not detected (MWU; p > 0.05)

CAP might induce membrane changes, such as loss of membrane symmetry or integrity, which ultimately results in loss of cell viability. To assess this, we exposed V79 fibroblasts cultured in Matriderm^®^ scaffolds to CAP for 1–4 min and determined the metabolic activity per V79 fibroblast as a measurement for viable cells. Compared to the unexposed control samples, exposure to CAP up to 3 min did not affect the viability of V79 fibroblasts. However, exposure to CAP for 4 min reduced the cell viability to 76% (Fig. 3).

### Effect of reactive species on cell viability and DNA mutations

The generation of CAP is accompanied by some heat. In addition, CAP commonly generates highly reactive molecules such as H_2_O_2_, O_3_ and NO_2_^−^ that could decrease pH of non-buffered solutions and induce oxidative and DNA damage. Therefore, we assessed these factors in relation to cell viability and DNA mutations.

**Fig. 4:**
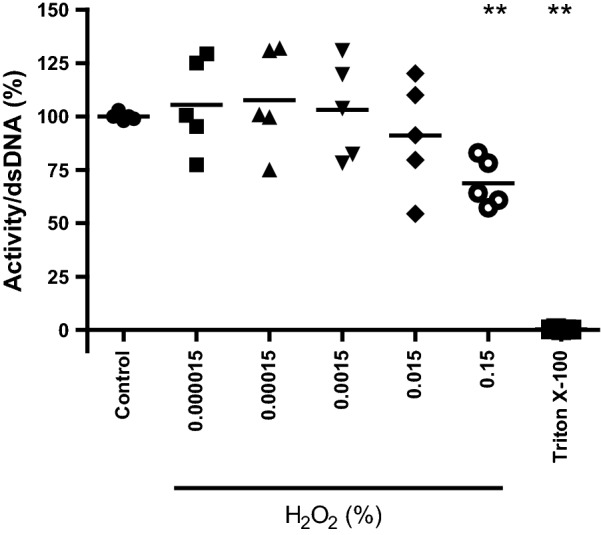
Susceptibility of V79 fibroblasts to H_2_O_2_. V79 fibroblasts in Matriderm^®^ were exposed to varying concentrations of H_2_O_2_ (0–0.15%; v/v) for 4 min. Subsequently, the metabolic activity per V79 fibroblast was determined. Results are expressed relative to the unexposed control. Data represent the means of five independent experiments performed in duplicate. Statistical differences compared to control samples are indicated: *p < 0.05; **p < 0.01; ***p < 0.001 (MWU)

We exposed V79 fibroblasts in Matriderm^®^ to heat from 50 to 70 °C water at a distance of 4 mm between sample and source and determined the cell viability. Additionally, we determined the effect of pH of the medium ranging from 6 to 3 and of H_2_O_2_ concentration in PBS ranging from 0 to 0.15% (v/v) on cell viability. Exposure to temperatures up to 70 °C or pH as low as 3 for 4 min did not affect the viability of V79 fibroblasts (data not shown). However, 0.15% (v/v) H_2_O_2_ reduced the cell viability to 69% (Fig. 4).

**Fig. 5:**
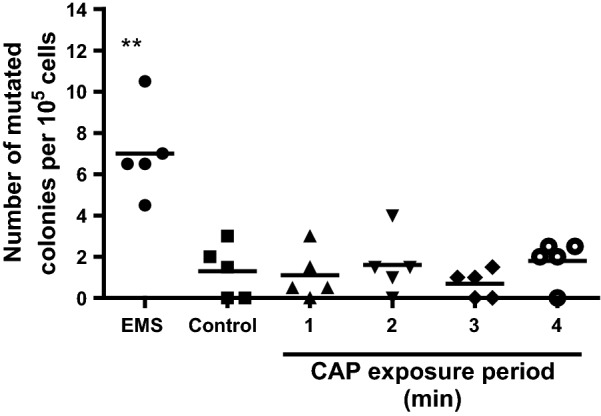
Effect of CAP on mutations in V79 fibroblasts. V79 fibroblasts in Matriderm^®^ were exposed to CAP for 1–4 min or EMS (positive control) and the number of mutated V79 fibroblasts was determined using a HPRT assay. As negative control the samples were washed using sterile saline. Results are expressed as the number of mutated colonies/10^5^ cells. Data represent the means of five independent experiments performed in duplicate. Statistical differences compared to the unexposed control samples are indicated: *p < 0.05; **p < 0.01; ***p < 0.001 (MWU)

Furthermore, we exposed V79 fibroblasts in Matriderm^®^ to CAP for 1–4 min and determined the mutation frequency by measuring the colony forming ability of the cells in the presence of cytotoxic 6-TG. Exposure of V79 fibroblasts in Matriderm^®^ to CAP resulted in 1–2 mutated colonies/10^5^ cells, independent of the exposure period (Fig. 5). This was comparable to the number of mutated colonies for the unexposed samples. Unlike CAP, EMS induced sevenfold higher numbers of mutations in V79 fibroblasts.

### Effect of repeated CAP exposure on wound healing in an ex vivo wound model

**Fig. 6:**
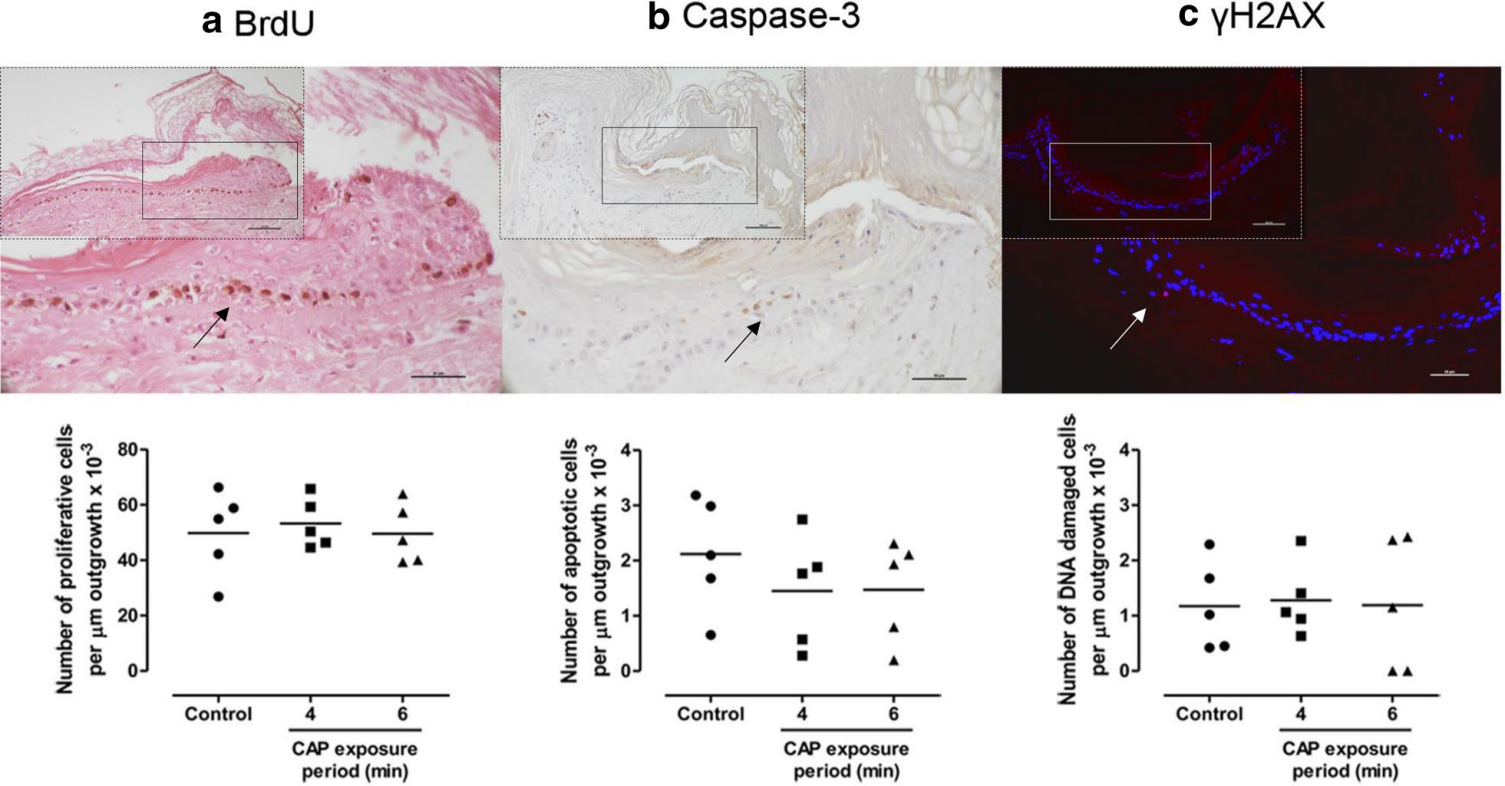
Effect of repeated exposure to CAP on ex vivo wound healing. During 2 weeks culture, BWMs were exposed four times (twice weekly) to CAP for 4 or 6 min or not exposed (negative control). Subsequently, the number of proliferative (**a**), apoptotic (**b**) and DNA damaged (**c**) cells per µm of newly formed epidermis (outgrowth) was determined using immunohistochemistry. The arrows indicate the positively-stained cells in the outgrowth of the unexposed BWMs (scale bars: 50 µm). This is also shown at a smaller magnification in the inset (scale bars: 100 µm). Data represent the means of five independent experiments performed in duplicate. No statistically significant differences were measured (Wilcoxon S-R; p > 0.05)

During 2 weeks of culture, ex vivo BWMs were exposed four times to CAP for 4 or 6 min to assess the effect on re-epithelialization, proliferation, apoptosis and DNA damage. Compared to the unexposed samples, exposure to CAP did not affect the re-epithelialization of BWMs. The re-epithelialization varied between 600 and 700 µm [12]. The additional safety assessments revealed that the number of proliferative, apoptotic and DNA damaged cells after exposure to CAP was not significantly different from those of the unexposed-control samples (Fig. 6).

### In vivo efficacy of CAP in a rat wound model

**Fig. 7:**
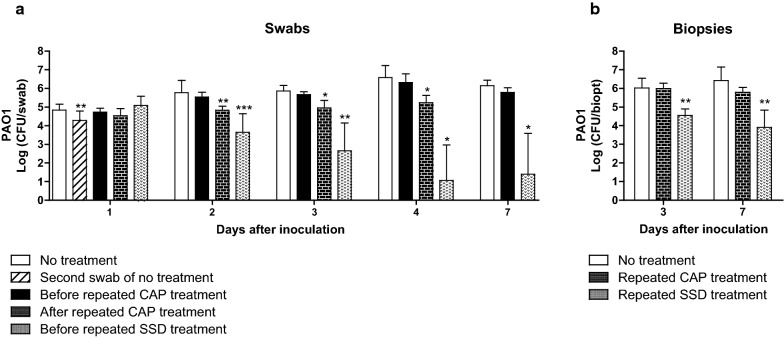
Antibacterial efficacy of CAP in a rat model. On the back of 24 rats, two excision wounds were inoculated with 10^7^ CFU PAO1 for 24 h. During 7 days, the wounds received a single CAP treatment (data not shown), repeated treatments on 4 consecutive days with CAP, 100 µL of 1% (wt/wt) silver sulfadiazine or no treatment. The bacterial load of the wounds was determined using daily swabs (**a**) or punch biopsies after euthanasia of rats on day 3 and day 7 (**b**). Results are expressed as the number of surviving bacteria in log10 (CFU/swab or biopt). Data represent the means of at least five samples. Statistical differences compared to CFU from swabs before treatment (Wilcoxon S-R) or biopsies of untreated wounds (MWU) are indicated: *p < 0.05; **p < 0.01; ***p < 0.001

Twenty-four hours after inoculation, just prior to treatment, the wound swabs showed a bacterial count of approximately 10^5^ CFU, which increased to 10^6^ CFU on day 7. Swabbing the same wounds twice resulted in approximately 0.5 log-reduction of the bacterial count on day 1 (Fig. 7a). A single CAP treatment on day 1 did not reduce the bacterial count significantly (data not shown). Repeated CAP treatment on 4 consecutive days resulted in a tenfold lower bacterial count of 1.7 × 10^5^ CFU on day 4, which increased to approximately 10^6^ CFU on day 7. Compared to the untreated wounds, CAP treatment increased the wound temperature with 3 °C on day 1 and with 5.9 °C on day 4. The discrepancy in temperature rise is most likely due to healing of the wounds. Furthermore, repeated treatment with SSD gradually and significantly reduced the bacterial count to 25 CFU PAO1 at day 7 but with high variation. Notably, SSD treatment was more than a 100-fold less effective against PAO1 which had penetrated the tissue (in biopsies) than against superficially located PAO1 (in swabs, Fig. 7a versus 7b).

## Discussion

CAP displays antimicrobial activity against a wide range of micro-organisms, such as bacteria [18]. It is efficacious regardless of the kind/species of bacteria and the antibiotic resistance level [19, 20]. This makes CAP an interesting therapy for the treatment of burn wound infections. CAP’s rapid mode of action against bacteria involves among others membrane lipid peroxidation, oxidative DNA damage and acidification [18], which might be harmful for human skin cells as well. Therefore, we assessed several safety aspects of CAP generated by the flexible sDBD in vitro*.* Our findings show that CAP exposure for 4–6 min did not induce mutations, apoptosis and DNA damage or affect the wound healing process, i.e. re-epithelialization and proliferation. The ability of CAP to induce mutations, apoptosis and DNA damage has been shown in a number of studies [21–25]. In fact, CAP could be a more potent mutagenesis tool compared to conventional mutagenesis systems [26]. Similar to our findings, several studies show that a relatively short treatment time with CAP has no mutagenic potential and does not induce apoptosis or DNA damage [27–29]. Additionally, Maisch et al. reports that CAP has no or a negligible effect on the viability of skin cells [29]. These findings indicate that CAP treatment can be used at specific settings for therapeutically safe applications.

Several studies show that CAP increases the temperature and decreases the pH of the exposed solution [30–33], which might be harmful for human skin (cells). Dobrynin et al. reports that toxic effects of CAP are related to the increase of the skin temperature, which is highly dependent on several factors such as the frequency of the discharge and the treatment time [34]. We found that 4 min exposure to heat or low pH alone did not affect the viability of the V79 fibroblasts. In contrast, the viability of V79 fibroblast was significantly reduced by 0.15% (v/v; equivalent to 49 mM) H_2_O_2_. However, H_2_O_2_ concentrations generated by CAP typically range from 0.3 to 1 mM [35] and these concentrations did not reduce the viability of V79 fibroblasts in our experiments. Next to H_2_O_2,_ also other reactive species such as O_3_, NO_3_^−^ and NO_2_^−^ are formed in the exposed liquid [36, 37]. The synergic interactions between the different reactive species could be responsible for the reduced viability of V79 fibroblasts. Such synergic interactions are also required to effectively eradicate bacteria [38–40]. Low pH or H_2_O_2_ alone were previously found to be insufficient to kill bacteria [35, 41]. Relatively high H_2_O_2_ concentrations of 490 mM or temperatures of 60 °C for a duration of 30 min were required to eradicate *P. aeruginosa* [41, 42].

Furthermore, we have shown that CAP generated by the flexible sDBD completely eradicated *P. aeruginosa *in vitro after a relatively short exposure period of 2 min*.* Additionally, CAP was efficacious against *P. aeruginosa* in ex vivo human skin models, whereby bacteria were effectively eliminated after 3 min exposure to CAP or after 6 min in BWM [12]. In view of these results, we anticipated that an exposure period of 6 min would result in an effective bacterial elimination in a rat wound infection model. However, CAP displayed a limited bactericidal efficacy against *P. aeruginosa* in this in vivo model. This suggests that wound environmental factors such as biofilm formation and wound exudate could have played a role in the limited efficacy of CAP in vivo. It was shown that bacteria in biofilms can be more tolerant against CAP [43–45]. Additionally, bacterial colonization and wound exudate could increase pH and/or introduce buffering effects to the wounds [46]. As a consequence, the bactericidal effect of CAP may be impeded because several reactive species are not generated at alkaline and buffered conditions [35, 47, 48]. For example, the concentration of free hydroxyl radicals from the decomposition of HNO_3_ is pH dependent, resulting in low radical concentrations at high pH [49].

Recently, Assadian et al. reported that plasma is safe but less effective in reducing the wound size or bacterial count as compared to current antimicrobial agents [50]. This is in agreement with our findings. Yet, several in vivo studies demonstrate an effective (but limited) elimination of bacteria using gas plasma treatments [51–53]. The successful elimination of bacteria using CAP is dependent on a number of factors such as the design of the device, treatment time, gas flow and composition, plasma power and frequency, the distance to the sample and environmental factors, such as the wound type, extracellular matrix or wound debris and exudate [54–56]. Possibly, it is more complex to achieve an effective bacterial elimination using CAP in the micro-environment of in vivo systems such as our rat wound infection model. We suggest to study CAP generated by this flexible sDBD device in combination with other antimicrobial or antibiofilm agents to combat bacteria. Previously, combination therapy of CAP and chlorhexidine for the disinfection of root canals resulted in a more effective elimination of bacteria than chlorhexidine or CAP alone [57]. Hence, combination therapy rather than monotherapy using CAP could potentially eliminate pathogenic bacteria more effectively in vivo.

## Conclusions

CAP did not induce mutations, apoptosis and DNA damage or affect the wound healing process in our in vitro and ex vivo (wound) models. Therefore, CAP can be considered a safe treatment option. CAP demonstrated a fast bactericidal effect in vitro, however, in our rat wound infection model CAP displayed a limited efficacy against PAO1.

## Data Availability

The datasets used and analyzed during the current study are available from the corresponding author on reasonable request.

## Abbreviations

BWM: Burn wound model
BrdU: 5-Bromo-2-deoxyuridine
CAP: Cold atmospheric plasma
CFU: Colony forming units
DMEM: Dulbecco's Modified Eagle Medium
DNA: Deoxyribonucleic acid
dsDNA: Double-stranded DNA
EDTA: Ethylenediaminetetraacetic acid
EMS: Ethyl methanesulfonate
FCS: Fetal calf serum
FBM: Fibroblast medium
HPRT: Hypoxanthine–guanine-phosphoribosyltransferase
LB: Luria Bertani
PBS: Phosphate-buffered saline
P/S: Penicillin/streptomycin
RPMI: Roswell Park Memorial Institute 1640
sDBD: Surface Dielectric Barrier Discharge
SSD: Silver sulfadiazine
6-TG: 6-Thioguanine
yH2AX: Gamma-H2A histone family member X
